# Global Quantitative Proteomics reveal Deregulation of Cytoskeletal and Apoptotic Signalling Proteins in Oral Tongue Squamous Cell Carcinoma

**DOI:** 10.1038/s41598-018-19937-3

**Published:** 2018-01-25

**Authors:** Sivagnanam Ananthi, Ch Naga Padma Lakshmi, Paul Atmika, Kumaraswamy Anbarasu, Sundarasamy Mahalingam

**Affiliations:** Department of Biotechnology, Bhupat and Jyoti Mehta School of Biosciences, Laboratory of Molecular Virology and Cell Biology, Indian Institute of Technology-Madras, Chennai, 600 036 India

## Abstract

Oral malignancies remain to have higher morbidity and mortality rates owing to the poor understanding of the carcinogenesis and the lack of early detection and diagnosis. The lack of established biomarkers for oral tongue squamous cell carcinoma (OTSCC) resulted in aggressive multi-modality management less effective. Here, we report for the first time that a panel of potential markers identified from tongue tumor samples using two-dimensional-differential-in-gel-electrophoresis (2D-DIGE). Our approach of combining 2D-DIGE with tandem mass spectrometry identified 24 candidate proteins including cofilins, myosin light chain family members, annexins, serpins, HSPs and tropomyosins, with significant differential expression in tongue carcinomas as compared with their matched adjacent normal tissues. The expression levels of the identified proteins were further validated in larger cohort of Indian samples using qPCR. Most of the differentially regulated proteins are involved in actin cytoskeletal dynamics, drug resistance, immune system, inflammation and apoptotic signalling pathways and are known to play critical role in oral tumorigenesis. Taken together, the results from present investigation provide a valuable base for understanding the development and progression of OTSCC. The validated panel of proteins may be used as potential biomarkers for early detection as well as in predicting therapeutic outcome of OTSCC.

## Introduction

Oral squamous cell carcinoma (OSCC) accounts for about 90% of malignant oral lesions and is widely recognized as the most frequently occurring malignant tumor of oral structures. Every year 500,000 new cases are diagnosed worldwide with only 50% survival rate over 5 years^1^. In India, the age standardized incidence rate of oral cancer is 12.6 per 100,000 persons^2^. The term ‘oral’ cancer includes cancers of the lip, tongue, gingiva, oral mucosa, oropharynx and hypopharynx^3^.

Oral tongue squamous cell carcinoma (OTSCC) is one of the most common sub-site of oral cancers. The trends in epidemiology of oral cancer in Asia in the past decade show tongue is the most frequently affected site^4^. Earlier studies report that India has a higher incidence of OTSCC as compared with other countries^5–8^. The incidence of OTSCC has shown an increasing trend in the population based cancer registry (PBCR) in India^9,10^. OTSCC has been observed to be associated with a poor prognosis and this may be due to the lack of biomarkers for early diagnosis as well as the faster disease progression compared to other oral sub-sites^9^. Clinical and histological features alone may not be sufficient to accurately predict tumor progression and outcome^11^. Although an increasing number of therapeutic strategies have become available during the last decade, more than one lakh people continue to face death due to aggressive oral cancers per year worldwide. Therefore, identification of molecular markers may advance precision diagnosis of OTSCC at very early stage and may help in understanding the biological process associated with aggressiveness of oral tongue cancer aiding rational drug design for better treatment and outcome^12,13^.

Proteomics, a study of the complete protein complements of the cell, is a promising approach for the identification of proteins that may be used as novel targets for therapeutic intervention and as biomarkers for early detection of cancers^14,15^. Proteomics approach has been successfully used for various tumors, and there is a large volume of data available on biomarkers in different tumor cells, tissues, and body fluids^14^. Recent studies have just attempted to understand the preliminary application of proteomics for the identification of biomarkers for OSCC^16–18^. Comparison of protein expression profiles between OSCC and normal cell lines or tissues has revealed replicable and significant changes in the expression levels of number of proteins, including some metabolic enzymes, modulators of signal transduction pathways, and oncoproteins^19,20^. However, a few of the identified proteins were found to vary between different cohorts, thus reflecting their variability or heterogeneity among populations. Interestingly, so far no report on the application of quantitative 2D-DIGE proteomic approach using oral tongue cancer tissues from Indian population. In the present investigation, we have profiled the protein map of human tongue tumor samples using 2D-DIGE coupled with tandem mass spectrometry and identified 24 differentially expressed proteins in tongue tumor samples. Further validation using qPCR analysis suggested that 16 proteins were up regulated and 8 proteins were down regulated in tongue cancer, which are significantly correlated with data obtained from quantitative proteomic analysis.

## Results

### Quantitative tongue cancer tissue protein profiling using 2D-DIGE analysis

**Figure 1:**
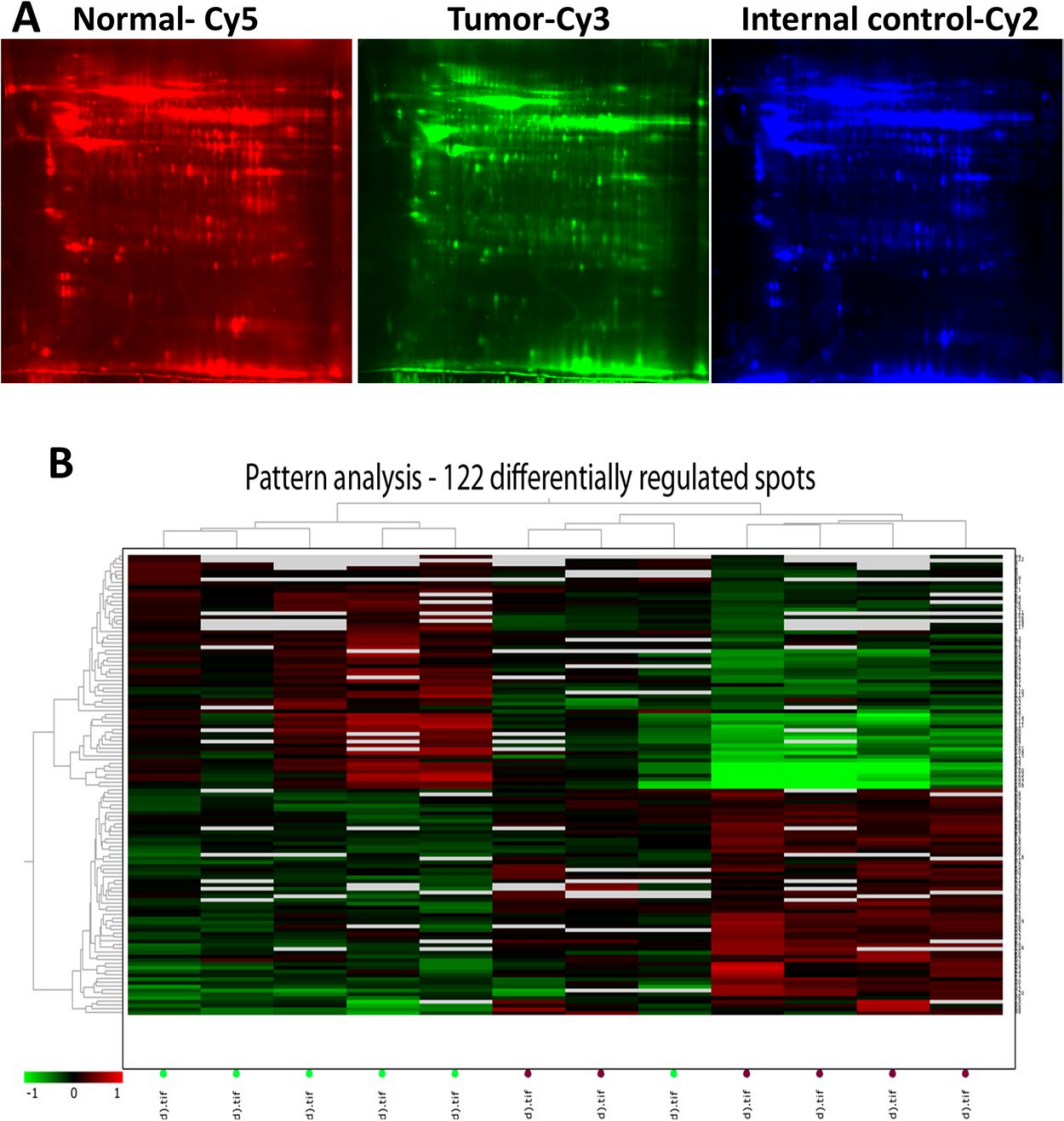
(**A**) Representative fluorescent protein profiles of a 2D-DIGE containing tongue normal sample labelled with Cy5, tumor sample labelled with Cy3 and pooled internal control labelled with Cy2. Tissue proteins were separated on IPG strip (pH 3–11) in the first dimension followed by 12.5% PAGE in the second-dimension electrophoresis. Images were captured using a Typhoon FLA9500 Variable Mode Imager. (**B**) Unsupervised hierarchical expression pattern of tongue tissue proteins. In ‘x’ axis, green dots indicate control samples and merun dots indicate tumor samples. Log-transformed normalized protein spot volumes were used to perform unsupervised hierarchical cluster analysis. Green indicates decreased expression; red indicates increased expression in the heat map.

Patient’s tissue sample details used in the current investigation are depicted in Table S1. Tissue extracts from tumor and normal tissue samples were prepared and analyzed by 2D-DIGE as described in Materials and Methods. The details of CyDyes labelling of tissue extracts are described in Table S2 and the representative images are shown in Fig. 1A. Typhoon FLA 9500 system was used to localize the protein spot positions in the 2D-DIGE images and the images were presented in Figure S1. CyDye labelled protein spots were analyzed using Differential In gel Analysis (DIA) module of the DeCyder software. Internal control was used to normalize the technical differences including laser exposure, CyDyes labeling and sample loading which will eventually lead to reproducible data acquisition. DIA module was used for the quantitation of protein (co-labeled with Cy3, Cy5 & Cy2 dyes) volume ratio within a single gel. The Cy2 labeling was used as internal control and the ratios of Cy5/Cy2 and Cy3/Cy2 labeling were determined using DIA module. Differentially expressed protein spots from six tumor samples with their respective normal were analyzed and the intensity of expression for all protein spots was represented in Table S3.

### Quantitative changes in protein expression profile between different tumor samples

Biological Variate Analysis (BVA) was used to analyze the differentially expressed proteins in tongue tumor and the adjacent normal tissue samples. Isoelectric focusing and second dimension analysis for all the samples were performed in a single experiment to eliminate gel to gel variations across all the samples. Stringent parameters were used to analyze the differentially expressed proteins and the following parameters are considered to confirm the differential expression of proteins, (a) minimum of 1.5 fold average fold ratio difference, (b) Cy2-internal control was detected in all experiments, (c) significant fold change (p < 0.05) and (d) spots were confirmed manually that they were true protein spots not artifacts. Analyses using above conditions, 122 proteins have been selected for further characterization. Interestingly, among these 122 proteins, 60 proteins were up regulated and 62 proteins were down regulated. Details of protein ID, average fold change ratio and statistical ANOVA values are described in Table S4 and Table S5, respectively. In tumor samples, up regulation of protein’s average fold change ratio range from 1.6–5.2 and down regulation of protein’s average fold change ratio range from-5.1– −1.5 fold.

### Hierarchical clustering

To further investigate the clinical importance of the protein expression profile identified in the current study, we performed hierarchical clustering analysis of 122 selected protein spots with DeCyder software. Figure 1B show that the heat map of fold variation in protein expression compared with internal control in six independent 2D-DIGE gels. Protein expression profiles were categorized into two distinct homogeneous clusters as tumors and normal. These results indicate that the differentially expressed proteins have a promising potential in differentiating the tumor samples from normal samples. Interestingly, similar protein expression profile pattern was observed across all tumor samples compared with normal samples.

### Identification of differentially expressed proteins by tandem mass spectrometry

**Figure 2:**
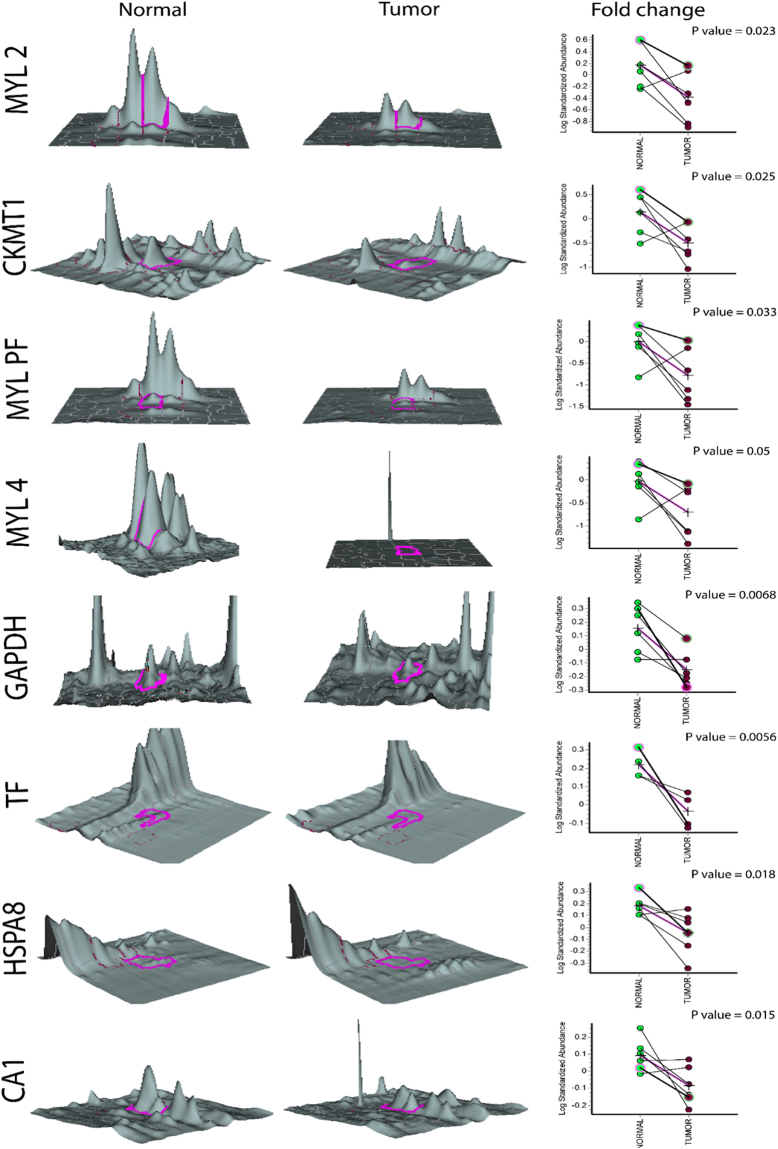
Expression pattern of down-regulated proteins in tongue tumor samples. Graphic views show the standardized log abundance of spot volume (y-axis) against the changes of proteins between the control and infected groups (x-axis) in all six samples. 3-D view of normal and tumor sample spots is also shown.

**Figure 3:**
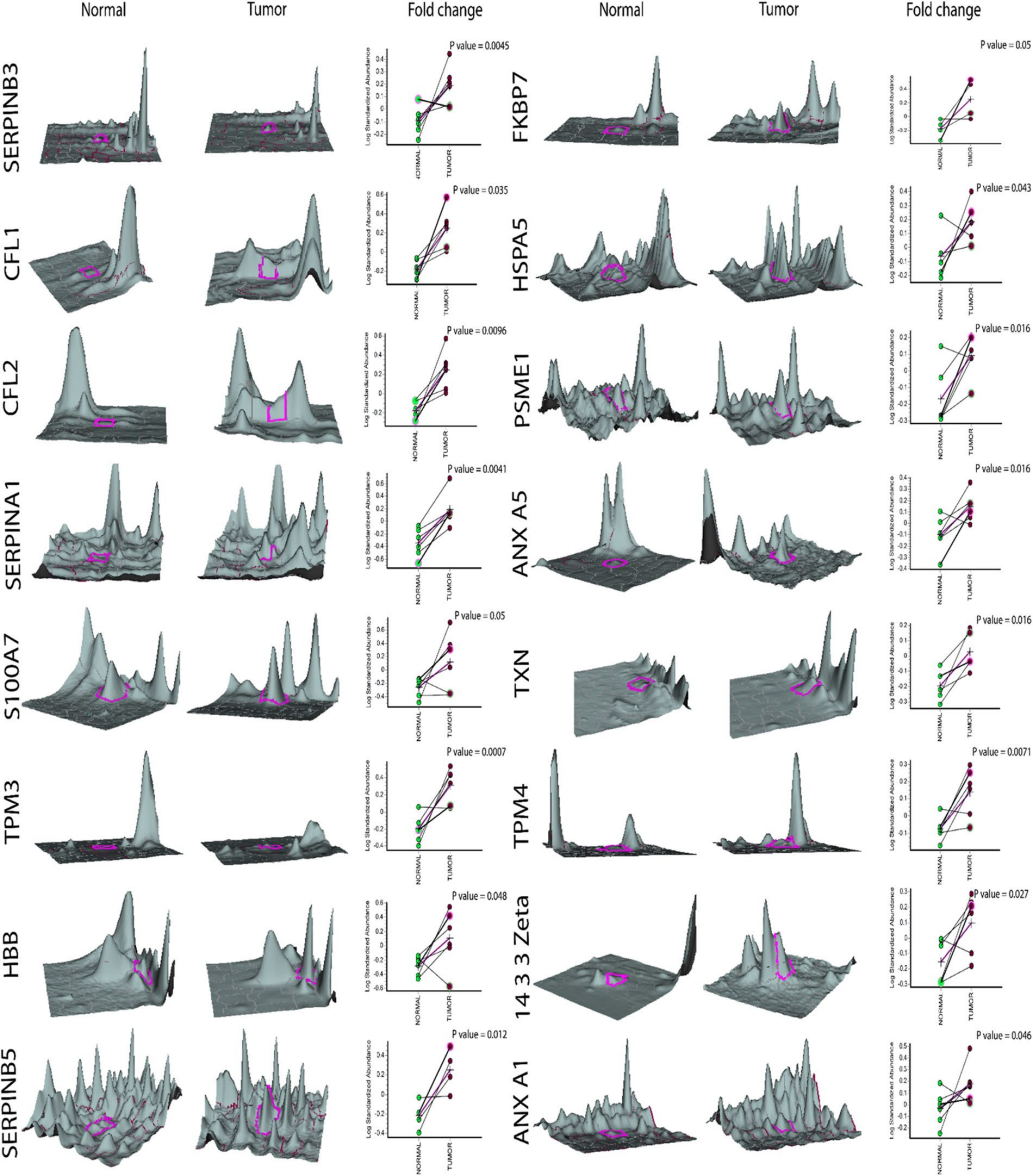
Expression pattern of up-regulated proteins in tongue tumor samples. Graphic views show the standardized log abundance of spot volume (y-axis) against the changes of proteins between the control and infected groups (x-axis) in all six samples. 3-D view of normal and tumor sample spots is also shown.

Tandem mass spectrometry analysis identified twenty-four differentially regulated proteins from tongue tumor samples (Figure S2) as described Materials and Methods. Table 1 summarizes the detail information about the proteins identified by nLC-MS/MS. Among the identified 24 protein candidates, 16 were upregulated and 8 were downregulated in tumor samples compared with matched normal tissue samples. The details about their average fold ratio and statistical significance values are represented in Table 2. Change of protein expression in terms of spot volume, 3-D representations of the spot volume and statistical significance between the tumor and normal tongue tissue samples are shown in Figs 2 and 3.

**Table 1:** List of the 24 differentially expressed proteins between tongue tumor and normal tongue samples identified by NanoLC-MS/MS.

Spot ID	Accession	Description	Score	Coverage	# Peptides	# PSMs	# AAs	MW [kDa]	Calc. pI
76	Q96A32	Myosin regulatory light chain 2, skeletal muscle isoform OS = Homo sapiens GN = MYLPF PE = 2 SV = 1 − [MLRS_HUMAN]	6190.5	94.67	36	2550	169	19	5.01
75	P10916	Myosin regulatory light chain 2, ventricular/cardiac muscle isoform OS = Homo sapiens GN = MYL2 PE = 1 SV = 3 − [MLRV_HUMAN]	4463.36	95.18	30	2096	166	18.8	5
288	P11142	Heat shock cognate 71 kDa protein OS = Homo sapiens GN = HSPA8 PE = 1 SV = 1 − [HSP7C_HUMAN]	3028.06	66.56	43	1659	646	70.9	5.52
284	P04083	Annexin A1 OS = Homo sapiens GN = ANXA1 PE = 1 SV = 2 − [ANXA1_HUMAN]	2815.72	85.55	46	1046	346	38.7	7.02
38	P08758	Annexin A5 OS = Homo sapiens GN = ANXA5 PE = 1 SV = 2 − [ANXA5_HUMAN]	2772	90	50	1368	320	35.9	5.05
275	P00915	Carbonic anhydrase 1 OS = Homo sapiens GN = CA1 PE = 1 SV = 2 − [CAH1_HUMAN]	2646.74	77.01	24	979	261	28.9	7.12
289	P11021	78 kDa glucose-regulated protein OS = Homo sapiens GN = HSPA5 PE = 1 SV = 2 − [GRP78_HUMAN]	2473.98	70.95	69	886	654	72.3	5.16
220	P23528	Cofilin-1 OS = Homo sapiens GN = CFL1 PE = 1 SV = 3 − [COF1_HUMAN]	2339.6	86.14	25	622	166	18.5	8.09
286	P29508	Serpin B3 OS = Homo sapiens GN = SERPINB3 PE = 1 SV = 2 − [SPB3_HUMAN]	1351.66	87.69	61	625	390	44.5	6.81
37	P67936	Tropomyosin alpha-4 chain OS = Homo sapiens GN = TPM4 PE = 1 SV = 3 − [TPM4_HUMAN]	1347.3	100	60	919	248	28.5	4.69
35	P63104	14-3-3 protein zeta/delta OS = Homo sapiens GN = YWHAZ PE = 1 SV = 1 − [1433Z_HUMAN]	1304.88	93.47	32	599	245	27.7	4.79
3	P68871	Hemoglobin subunit beta OS = Homo sapiens GN = HBB PE = 1 SV = 2 − [HBB_HUMAN]	1225.3	80.27	13	573	147	16	7.28
305	P36952	Serpin B5 OS = Homo sapiens GN = SERPINB5 PE = 1 SV = 2 − [SPB5_HUMAN]	993.08	72.53	36	394	375	42.1	6.05
179	P12532	Creatine kinase U-type, mitochondrial OS = Homo sapiens GN = CKMT1A PE = 1 SV = 1 − [KCRU_HUMAN]	837.7	57.31	34	301	417	47	8.34
220	Q9Y281	Cofilin-2 OS = Homo sapiens GN = CFL2 PE = 1 SV = 1 − [COF2_HUMAN]	770.28	57.23	4	1	15	268	166
308	Q06323	Proteasome activator complex subunit 1 OS = Homo sapiens GN = PSME1 PE = 1 SV = 1 − [PSME1_HUMAN]	744.05	72.69	27	344	249	28.7	6.02
287	P02787	Serotransferrin OS = Homo sapiens GN = TF PE = 1 SV = 3 − [TRFE_HUMAN]	570.38	44.99	43	259	698	77	7.12
2	P31151	Protein S100-A7 OS = Homo sapiens GN = S100A7 PE = 1 SV = 4 − [S10A7_HUMAN]	472.07	92.08	14	313	101	11.5	6.77
221	Q71V99	Peptidyl-prolyl cis-trans isomerase OS = Homo sapiens PE = 2 SV = 1 − [Q71V99_HUMAN]	352.84	71.95	15	363	164	18	7.9
86	P12829	Myosin light chain 4 OS = Homo sapiens GN = MYL4 PE = 1 SV = 3 − [MYL4_HUMAN]	286.17	87.31	21	239	197	21.6	5.03
280	P04406	Glyceraldehyde-3-phosphate dehydrogenase OS = Homo sapiens GN = GAPDH PE = 1 SV = 3 − [G3P_HUMAN]	214.78	73.13	23	91	335	36	8.46
18	P10599	Thioredoxin OS = Homo sapiens GN = TXN PE = 1 SV = 3 − [THIO_HUMAN]	164.01	50.48	9	157	105	11.7	4.92
177	P01009	Alpha-1-antitrypsin OS = Homo sapiens GN = SERPINA1 PE = 1 SV = 3 − [A1AT_HUMAN]	74.17	52.63	22	29	418	46.7	5.59
371	P06753	Tropomyosin alpha-3 chain OS = Homo sapiens GN = TPM3 PE = 1 SV = 1 − [TPM3_HUMAN]	60.22	69.37	33	57	284	32.8	4.72

The table shows the MS output list of the 24 proteins identified that were significantly up- or down-regulated in tongue tumor versus normal tongue. Spot ID, accession number, protein name, score, % coverage, number of covering peptides, peptide spectrum match (PSM), aminoacid number (AA), molecular weight (calc MW) and calculated isoelectric point (calc. pI) are described.

**Table 2:** List of 24 differentially regulated proteins describing the name, average fold ratio, statistical significance value and regulation status obtained from Biological Variate analysis in DeCyder Software.

S.No	Name	Av. Ratio	1-ANOVA	Regulation in tumor
1	CKMT1	−4.62	0.025	DOWN
2	MYL PF	−3.75	0.033	DOWN
3	MYL 4	−3.53	0.05	DOWN
4	GAPDH	−2.06	0.0068	DOWN
5	TF	−1.78	0.0056	DOWN
6	HSPA8	−1.62	0.018	DOWN
7	CA1	−1.5	0.015	DOWN
8	MYL2	−4.65	0.023	DOWN
9	SERPINA1	3.6	0.0041	UP
10	S100A7	3.37	0.05	UP
11	TPM3	3.31	0.0007	UP
12	HBB	3.1	0.048	UP
13	SERPINB5	3.08	0.012	UP
14	FKBP7	3.02	0.035	UP
15	YWHAZ	1.8	0.027	UP
16	SERPINB3	1.98	0.0045	UP
17	HSPA5	1.74	0.043	UP
18	PSME1	1.73	0.016	UP
19	ANXA5	1.72	0.016	UP
20	TXN	1.71	0.016	UP
21	TPM4	1.7	0.0071	UP
22	CFL1	3.02	0.035	UP
23	CFL2	2.89	0.00096	UP
24	ANXA1	1.64	0.046	UP

### Validation of differentially expressed proteins by qPCR

**Figure 4:**
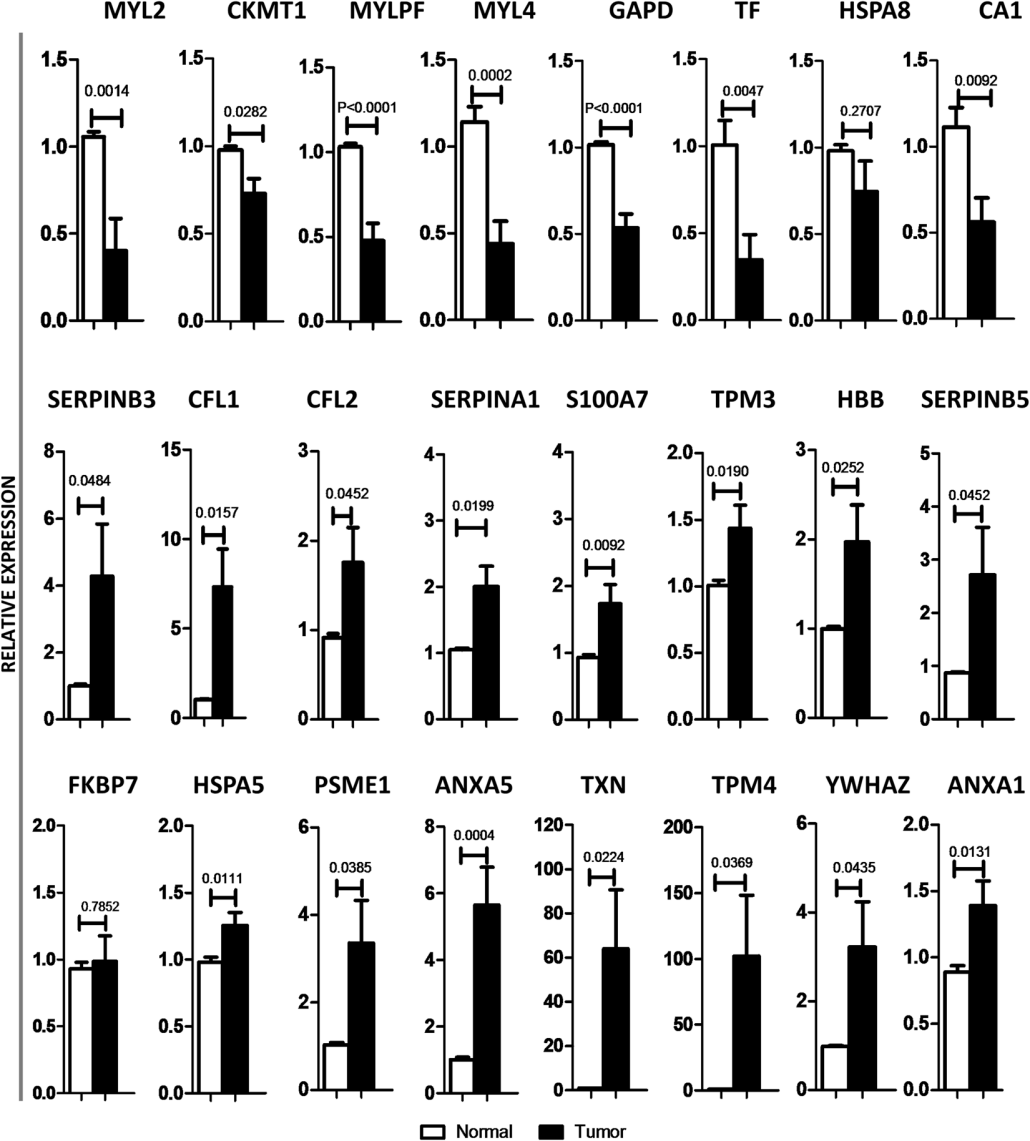
Validation of differential expressed genes by qPCR analysis. Expression pattern of twenty four significantly altered candidate genes were analyzed in tongue tumor and normal tissue samples using qPCR.

To confirm the differential expression of proteins that are identified by 2D-DIGE followed by nLC-MS/MS, we performed qPCR analysis. Among 24 differentially regulated proteins, 8 were downregulated and 16 were upregulated in tumor samples compared with respective normal and apparent normal tissues (Fig. 4) which is in agreement with 2D-DIGE analysis. MMP9 gene expression was determined for all the samples and it served as internal control for differentiating tumor samples from normal samples (Figure S3).

### Functional classification of identified proteins and biological network analysis

**Figure 5:**
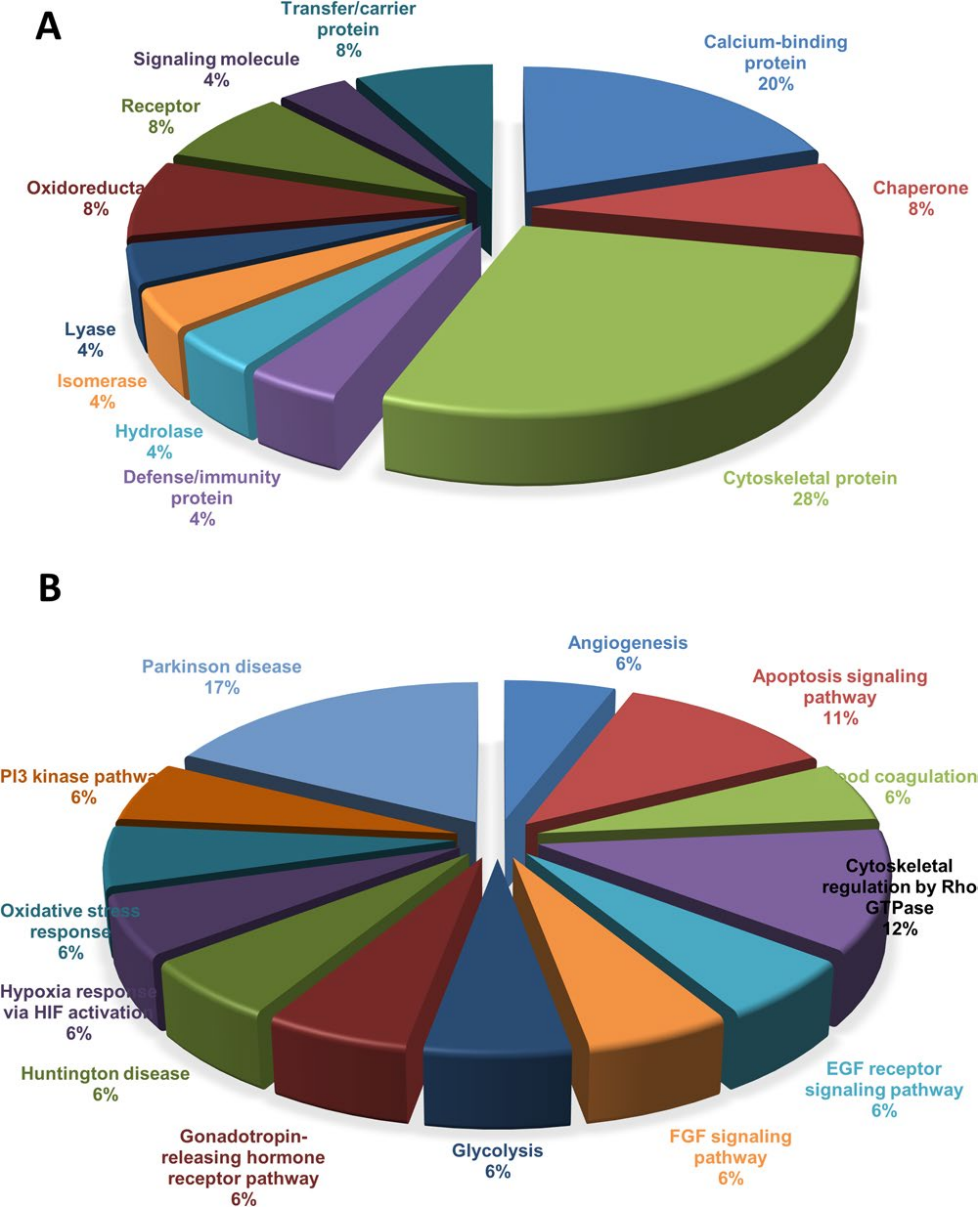
Protein classification and Pathway analysis. PANTHER database was used for protein classification (**A**) and pathways (**B**) analysis for possible functions for 24 differentially regulated proteins.

We next performed **P**rotein **AN**alysis **TH**rough **E**volutionary **R**elationships (PANTHER)^21^ analysis to gain better understanding on the functions of all the differentially expressed proteins identified by nLC-MS/MS. All differentially expressed proteins were categorized into three groups based on their cellular localization, molecular function, and biological process (Figure S4A–C). Among the 24 differentially expressed proteins, 21% proteins were found to be involved in cellular process, 16% were involved in biological regulation & metabolic process and 14% in multicellular organismal process. About 7% involved in response to stimulus and immune process. In addition, most of the identified proteins were related to cellular part, 29% related with organelle and few related with membrane and macromolecular complex. With respect to the molecular function of the identified proteins, most of them have binding activity, followed with structural molecule and catalytic activity. Most of them were calcium binding proteins and cytoskeletal proteins (Fig. 5A). Few were involved in channel regulation, signal transduction, and transporter activity. Accumulating all the functional categories, it is very evident that all these differentially expressed proteins play a critical role in tumorigenesis, having their major involvement with metabolic process and other biological regulations including glycolysis, cytoskeletal regulation by Rho GTPase, apoptosis signaling pathway, p53 pathway, angiogenesis, hypoxia response pathway and MAPK pathway (Fig. 5B).

**Figure 6:**
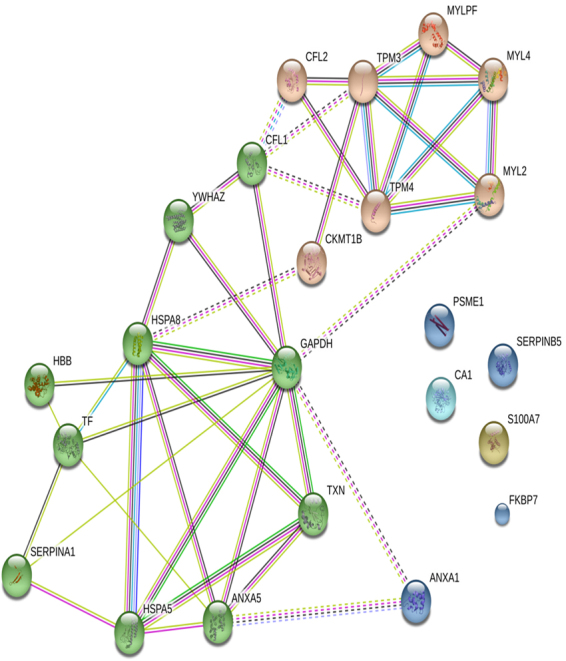
Protein-protein interaction analysis performed for 8 down regulated proteins and 16 up regulated proteins using STRING database. Cluster analysis using k means clustering was performed and highlighted as two cluster.

The protein-protein interaction analysis was also performed for all these 24 differentially regulated proteins using STRING database^22^. Analysis revealed that among 24, five proteins stand as individual players and are not reported to interact with other proteins identified in the present investigation. Remaining proteins interact either directly or indirectly through other proteins networks (Fig. 6). Five protein networks were observed, cofilin isoforms and myosin light chain proteins interacting with tropomyosins, heat shock proteins forming a network connecting with other protein networks such as metabolism and annexin proteins (Fig. 6). Results in Figure S5A and B describes the novel protein interaction networks for downregulated and upregulated proteins, respectively. Cluster analysis was performed for all the interaction networks by K means clustering and found that are grouped into two major clusters, one with all cytoskeletal proteins including cofilin 2, myosin light chain family members and tropomyosin. Other major cluster includes all heat shock proteins, inflammatory proteins, SERPIN family proteins and annexin.

## Discussion

Differential proteomic analysis of cancer tissues compared with normal counterparts enables the definition of specific proteomic profile, characterizing the pathophysiology of tumorigenesis process. Our study, for the first time, applies a 2D-DIGE coupled with tandem mass spectrometry approach for the analysis of tongue tumor tissues and identified protein signatures that are differentially expressed in tongue tumor samples. Previous studies have focused on genomic based analysis on tongue carcinoma^10^. Despite permitting a comprehensive analysis of mRNA transcripts, these studies may not provide information on the observed mRNA modulation corresponds to a consequent modulation of the encoded proteins. Indeed, steady-state transcript abundance only partially predicts protein levels in various systems^23^. Recent study used the conventional 2D electrophoresis^17^ rather than the more sensitive and reliable 2D-DIGE based quantitative approach and identified a panel of 12 proteins in tongue cancer, which were not validated with normal tissue samples in the same gels that could lead to biased conclusions owing gel-to gel variations.

Conversely, 2D-DIGE technique enables direct comparison of protein profile between tumor and normal samples on the same 2D gel, thus reducing technical variability which could affect the expression pattern of proteins. Moreover, the introduction of fluorescent dyes to differentially mark the control and diseased samples exponentially increases the sensitivity of detection. Finally, the use of an internal control, consisting of a mixture of equal amounts of all the samples to be analyzed, allows precise quantification of differential protein expression, regardless of the number of matched gels and arbitrary matching of normal and tumor samples in each gel. The proteomics approach has been widely used to identify potential biomarkers for early detection of various tumors (prostate, breast, colon). In particular, this method has a relevant clinical impact when biomarkers identified in the tumor tissue samples, also differentially expressed in the bloodstream^24^.

In the current study, the proteomic profile of tongue tumor tissue samples was compared with matched normal tongue samples and observed 24 proteins were significantly deregulated. The qPCR analysis clearly suggested that these genes are differentially expressed in tongue tumor tissue samples in comparison with normal counterparts which is in accordance with proteomic analysis. Among 24 candidates, 8 proteins including Myosin light chain family members (MYLPF, MYL4 and MYL2), creatinine kinase, serotransferrin, heat shock protein A8, carbonic anhydrase 1 and Glyceraldehyde-3-phosphate dehydrogenase were significantly downregulated in tongue tumor tissues. In contrast, 16 proteins including annexins (ANXA1 and ANXA5), serpins (SERPA1, SERPB3 and SERP5), tropomyosins (TPM3 and TPM4), cofilins (CFL1 and CFL2), hemoglobulin, Peptidyl-prolyl cis-trans isomerase, Proteasome activator complex subunit, thioredoxin, 14-3-3 zeta, S100A7, heat shock protein A5 were significantly upregulated in tongue tumor samples compared with normal tissue samples. Correlation analysis of the identified target genes with available data base (BioXpress) for head and neck squamous cell carcinoma reveals that 16 genes show a strong positive correlation (Figure S6A) whereas 6 genes showed strong negative correlation (Figure S6B) suggest that these 6 genes might be forming a population specific biomarkers. Taken together, these analyses highlight the importance of biomarker based population screening and their potential use in future therapeutic strategies for better management of tongue cancers.

Analysis of reported cellular functions for the 24 differentially regulated proteins indicated that cell survival (17 proteins) and cytoskeletal remodeling (7 proteins) pathways are significantly altered in OTSCC. Among the 17 proteins modulating cell survival cascade, CKMT1, GAPDH and CA1 were known to have a pro-apoptotic role whereas SERPIN (A1, B3, B5), YWHAZ, PSME1 and TXN negatively regulate the apoptotic pathways. In addition, HBB, S100A7, TF impinge on cell proliferative pathways while FKBP7, HSP (A5, A8) and ANX (A1, A5) confer resistance to chemotherapy, thereby may be contributing to poor prognosis in OTSCC.

Evasion of apoptosis, being a major hallmark of cancer, is one of the emerging strategies for targeted treatment against neoplastic progression^25,26^. Deregulated expression of pro- and anti-apoptotic proteins could ultimately result in prolonged cell survival by subversion of apoptotic pathways. Results from the present study suggest a significant downregulation of pro-apoptotic proteins - CKMT1, GAPDH and CA1 and upregulation of anti-apoptotic proteins – SERPIN (A1, B3, B5), YWHAZ, PSME1 and TXN in tongue cancer samples as compared to matched normal samples suggest their role in cell survival during tumorigenesis. Although previous reports^27–34^ suggest their functional significance in promoting EMT, involvement in cellular signaling, ability in targeting telomerase complex and protecting cancer cells from death signals, very limited information is available regarding their putative roles in tongue tumorigenesis.

Another notable hallmark of cancer is the limitless replicative potential and ability of the cancer cells to maintain a sustained proliferative signaling^25,26^. It is well-established that the deregulation of key players controlling proliferative signaling pathways eventually leads to malignant progression and thereby a sustained poor outcome. In the present study, significant upregulation of HBB, S100A7 and TF was observed, which is consistent with reports on their pro-proliferative activity in various cancers^35–42^. A major reason behind the inevitable attenuated response of cancer patients to chemotherapy is the property of multi-drug resistance acquired by cancer cells. The present work has witnessed an increased expression of FKBP7, HSPA5 and ANX (A1, A5) reported to act as key players in conferring resistance to drug treatment^43–48^. However, the role of these molecules in neoplastic progression of oral cancer is yet to be explored. One of the major obstacles in cancer related deaths is due to metastasis. The metastatic cascade requires an alteration of the cellular machinery involved in cytoskeletal remodeling. This enables the cells to move from their primary site and colonize in the distant site. Thus, to define the function of proteins involved in cytoskeletal reorganization is crucial to understand the mechanism behind the malignant progression of cancers. While cofilins are reported to modulate G-actin/F-actin ratio by binding directly to the actin filaments and affect the actin dynamics^49^, the tropomyosin family of proteins form an important component of intermediate filaments and help in the contraction of skeletal and smooth muscle cells or maintaining the cytoskeletal stability in non-muscular cells^50^. In contrast, the myosin light chain family of proteins are known to regulate myofilament activation via phosphorylation by Ca^2+^dependent myosin light chain kinase^51^. The present study identified cofilins (CFL1 and CFL2) and tropomyosin family proteins (TPM3 and TPM4) are significantly upregulated, in contrast, myosin family proteins (MYL2, MYL4 and MYLPF) were downregulated in tongue tumor samples as compared to normal samples. Taken together, deregulation of these proteins plays pivotal roles in stress fiber formation, cell motility and cytokinesis, which may be associated with the invasion and metastasis of tongue carcinoma.

In conclusion, the proteomic expression profiling of tongue cancer samples using 2D-DIGE based approach coupled with mass spectrometry led to the identification of a panel of twenty-four dysregulated proteins. Further validation by qPCR confirmed their differential regulation at transcriptional level. Hence, these results suggest their potential role as biomarkers for oral tongue squamous cell carcinoma. Further studies with larger cohorts of tongue cancer patient samples at different stages are required to confer diagnostic and prognostic significance to these biomarkers with respect to OTSCC.

## Methods

### Patients

The study was approved by the Institutional Ethical Committee (Ref No: IEC/2016/01/SM-5/15) of Indian Institute of Technology Madras, Chennai, India. Informed consent was obtained from all patients. Totally 110 tissue samples were used in the present investigation and the samples details are described in Table S1. All the samples were obtained from National Cancer Tissue Biobank (NCTB), Indian Institute of Technology Madras, Chennai. Tumor and normal tissue samples were snap frozen and stored at liquid nitrogen until used for protein and RNA extraction. Adjacent normal samples were used as controls in discovery phase and apparent normal tissue samples were used for validation phase. All methods were performed in accordance with the guidelines and regulations approved by Institutional ethics committee of Indian Institute of Technology Madras, Chennai, India.

### Proteins Labeling with CyDyes

Tissue extracts were prepared by grinding the samples in chilled mortar and pestle with liquid nitrogen and dissolved in lysis buffer (7 M Urea, 2 M Thiourea, 4% CHAPS, 20 mM PMSF & 20 mM DTT). Tissue samples were further lysed by sonication for 10 min and centrifuged at 12,000 rpm for 15 min at 4 °C. Tissue extracts were collected and protein estimation was performed using Bradford method before being aliquoted and stored at −80 °C for further analysis.

For protein labelling (Lysine labeling), Cydyes were reconstituted (400 pmol/ml) with anhydrous N,N-dimethylformamide (Sigma Aldrich chemical co. USA) and stored at −20 °C. The dyes were further diluted (100 pmol/ml) and used immediately. Tissue proteins (tumor and normal) were labeled individually with Cy5 and Cy3 dyes. For internal control, tissue extracts containing equal amount of proteins were mixed and labeled with Cy2. Cy5 or Cy3 was added to the tissue extracts containing 30 μg proteins and incubated at 4 °C in dark for 30 min. Labelling was stopped by adding 10 mM lysine (2 μl) followed by incubation on ice for 10 min. Equal volume of Cy5 and Cy3 labeled tissue extracts were mixed with Cy2 labelled extracts followed by the addition of lysis buffer containing DDT (100 mM) and IPG buffer (2% v/v) and further incubated at room temperature for 10 min. Rehydration buffer [urea (7 M), thiourea (2 M), IPG buffer (1%), DTT (50 mM), CHAPS (4%), and bromophenol blue] was added to the samples and adjusted the final volume up to 340 μl. Dye swapping condition was also adapted to avoid the preferential labeling. Table S2 summarizes the labeling methods for the 2D-DIGE experiments.

### Two-dimensional Difference In gel electrophoresis (2D-DIGE) and Image analysis

Two-dimensional difference in gel electrophoresis was performed as described elsewhere [53] with minor modifications. IPG strips (18 cm) of pH 3–11 (GE Healthcare, Sweden) were used for isoelectric focusing. Samples were loaded on IPG strips and focused for a total of 82,000 Vhs at a constant temperature (20 °C) under linear voltage ramp after an active IPG rehydration at 30 V in a IPGPhor III (GE Healthcare, Sweden) system with following parameters, 500 V step-n-hold for 1 h, 1000 V gradient for 1 h, 8000 V gradient for 3 h and 8000 V step-n-hold for 8 h. After isoelectric focusing, all the strips were equilibrated with buffers containing DTT (2%) followed by 2.5% iodoacetamide before the focused proteins were resolved in second dimension with 12.5% PAGE for 6 hours in dim light. Typhoon FLA 9500 Variable Mode Imager (GE Healthcare, Uppsala, Sweden) was used for scanning the Cy2, Cy3 & Cy5 images. Scanned gel images were imported to the DeCyder Differential Analysis Software (version 7.0) and the spot volume was standardized and estimated as the differential ratio across the actual volume of the particular spot compared with the whole volume for all spots. All gel images (Cy5 and Cy3) within a set of experiment were intrinsically linked with the internal standard (Cy2) on each gel. Spot intensity analysis within the same gel was performed by DeCyder differential in-gel analysis (DIA). DeCyder biological variation analysis (BVA) was used for spot intensity analysis across various experiments. Differences in the spot intensity ratio for the significantly altered proteins was expressed as “fold” change. Positive values show the up regulation and negative values show the down regulation of protein spot intensity in the tumor samples. On every gel, all the spots have been normalized with the matching spot on the pooled internal standard. Significant difference in the average spot volume for all visible spot pairs between the tumor and normal set of samples was estimated using ANOVA. Reciprocal dye labeling method was adapted to normalize the variations in labeling.

### In gel trypsin digestion and nLC-MS/MS analysis

Total tongue tissue proteins (250 ug) were analyzed by first dimension separation with 18 cm IPG strips of pH 3–11 followed by second dimension PAGE analysis as described above. Separated proteins were processed for colloidal coomassie blue G-250 staining and protein spots of interest were subjected to in-gel trypsin digestion followed by mass spectrometry analysis. Briefly, prior to destaining, gel pieces were washed with milli Q H2O and gel pieces containing proteins were completely destained by washing with destaining solution containing 50% acetonitrile and 25 mM ammonium bicarbonate (NH4HCO3). Destained gel pieces were completely dehydrated in 100% acetonitrile (ACN) for 10 min and dried under vacuum for 30 min. The gel pieces were rehydrated/trypsinized with 5 μl of trypsin buffer (10 mM NH4HCO3 in 10% ACN) containing 400 ng of trypsin (Sigma Aldrich, USA) on ice for 30 min and overlayed with 25 μl of buffer (40 mM NH4HCO3 in 10% ACN) and incubated at 37 °C for 16 h. After incubation, peptides were extracted twice with 25 μl of 0.1% trifluoroacetic acid (TFA) in 60% ACN by sonication (10 min) followed by 20 μl of 100% ACN. Extracted peptides were dried under vacuum for 90 min and stored at 4 °C [33]. For LC-MS/MS analysis, dried peptides were dissolved in peptide resuspension solution (0.1% TFA in 5% ACN) and desalted using C18 zip tips (Merck Millipore, USA) as per the manufacturer’s protocol. Zip tip purified peptides were analyzed using nano-RPLC (Thermo Scientific, USA) coupled with an Orbitrap Elite Mass spectrometer (Thermo Scientific, USA). Peptide mixtures were dissolved in 2% ACN with 0.1% of formic acid and loaded onto a guard column (EASY-spray column pepMap®RSLC, C18, 5 μm, 100 μm × 2 cm Thermo Scientific, CA). Purified peptides were released into a C18 capillary column (100 μm × 10 cm) and separated using a linear gradient solvent system (5–100% ACN) for 80 min at a flow rate of 300 nl/min. Peptides were ionized by positive mode electrospray with an ion spray voltage of 1.9 kV. MS data were acquired in positive ion mode over mass range m/z 350–4000 Da using Xcalibur software (version 2.2.SP1.48) (Thermo Scientific, USA). MS data were analyzed using Proteome Discoverer software v.1.4 (Thermo Scientific, USA) using sequest algorithm. Following parameters were used for protein identification: enzyme-trypsin, missed cleavages - 2, precursor mass tolerance - 10 ppm, fragment mass tolerance - 0.5 Da, carbamidomethylation of cysteine was given as fixed modification whereas methionine oxidation, N-terminal acetylation and phosphorylation (S, T, Y) as variable modifications^52^.

### Quantitative Real time PCR

RNA was isolated from tongue normal (20) & tumor (30) samples using RNeasy kit (Qiagen, USA) according to manufacturer’s instructions. Quantitative Real Time PCR (qPCR) analysis was carried out using SYBR-Green mix (Takkara, USA) on Realplex cycler (Eppendorf, Germany) for measuring the mRNA expression of 24 genes and are listed in Table 1. The primer sequences used for qPCR analysis are listed in Table S6. Universal thermal cycling conditions were used as follows: 3 minutes at 95 °C, 40 cycles of denaturation at 95 °C for 30 seconds, annealing and extension at 58.1 °C, 60 °C for 20 seconds each respectively. The comparative threshold cycle (Ct) method was used to calculate fold change. Beta actin was used as a reference control to normalize the expression values. Triplicate reactions were performed for each gene and the relative gene expression level was calculated using 2-ddct method.

### Functional classification of proteins and pathway analysis

The gene symbols of the differentially expressed proteins were given as input to PANTHER database for functional classification and pathway analysis. STRING (www.string.db.org) was used for protein network construction. The interactions include direct (physical) and indirect (functional) associations that are derived from four sources namely genomic context, high throughput experimentation, previous knowledge, conserved co-expression. Quantitatively integrated interaction data from these sources were used to derive the integration maps.

### Correlation analysis

Expression data were retrieved from BioXpress database^53^ for target genes identified in the present study. The expression data was subjected to correlation analysis against Average Fold-Ratio obtained from 2D-DIGE gels spot intensity as derived from DeCyder analysis using GraphPadPrism 5 (GraphPad software, USA). Pearson correlation coefficient (r-value) was calculated and represented in the scattered plots.

### Statistical analysis

Relative quantity of stained protein spots compared with the internal standard spots were analyzed by DeCyder Difference In-gel Analysis (DIA) and DeCyder Biological Variation Analysis (BVA) softwares (GE Healthcare, Sweden). Student’s t-test was used to calculate statistically significant differences between groups. P < 0.05 was considered statistically significant. For qPCR analysis, Student’s t-test was performed to calculate the statistical significance of differential expression levels between tumor and normal samples.

## Electronic supplementary material

Supplementary information
