# Supplementary material for: Global Quantitative Proteomics reveal Deregulation of Cytoskeletal and Apoptotic Signalling Proteins in Oral Tongue Squamous Cell Carcinoma
Source: Sci Rep. 2018 Jan 25;8:1567. doi: 10.1038/s41598-018-19937-3 (PMC5785498; doi:10.1038/s41598-018-19937-3)
Supplement: Supplementary file 1 — Supplementary information [file 41598_2018_19937_MOESM1_ESM.pdf]

Global Quantitative Proteomics reveal Deregulation of Cytoskeletal and Apoptotic Signaling Proteins in Oral Tongue Squamous Cell Carcinoma

Sivagnanam Ananthi, Ch Naga Padma Lakshmi, Paul Atmika, Kumaraswamy Anbarasu,

Sundarasamy Mahalingam *

Figure S1 :

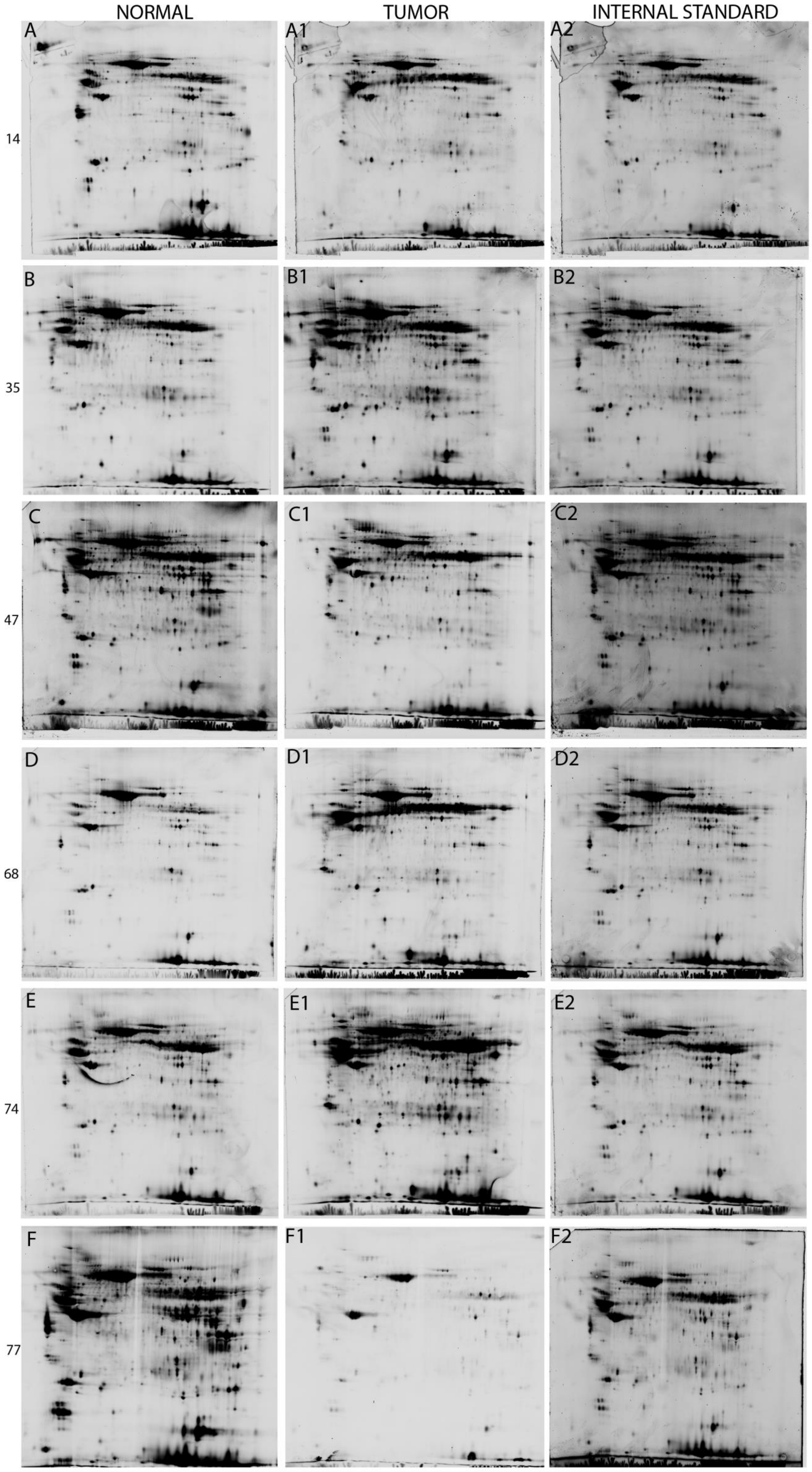
NORMAL
TUMOR
INTERNAL STANDARD
A
A1
A2
14
B
B1
B2
35
C
C1
C2
47
D
D1
D2
68
E
E1
E2
74
F
F1
F2
77

Figure S2

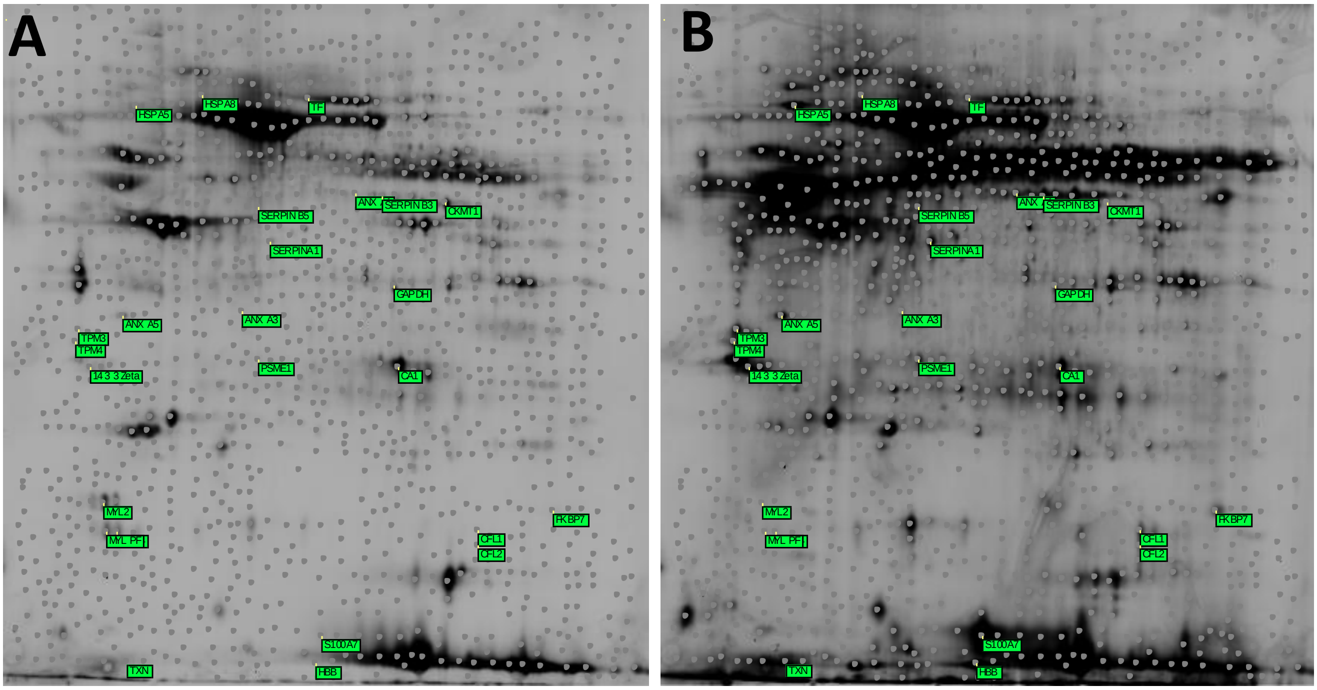

A
B
HSPA5
HSPA8
TF
SERPINB3
CKMT1
SERPINB5
SERPINA1
GAPDH
ANXA5
ANXA3
TPM3
TPM4
14 3 3 Zeta
PSME1
CA1
MYL2
FKBP7
CFL1
CFL2
S100A7
TXN
HBB

Figure S3

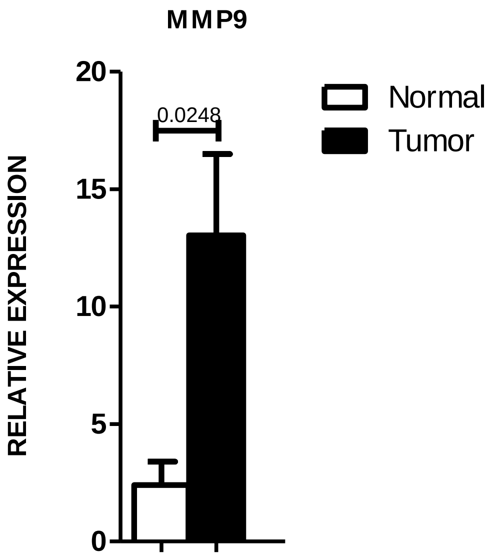

Figure S4

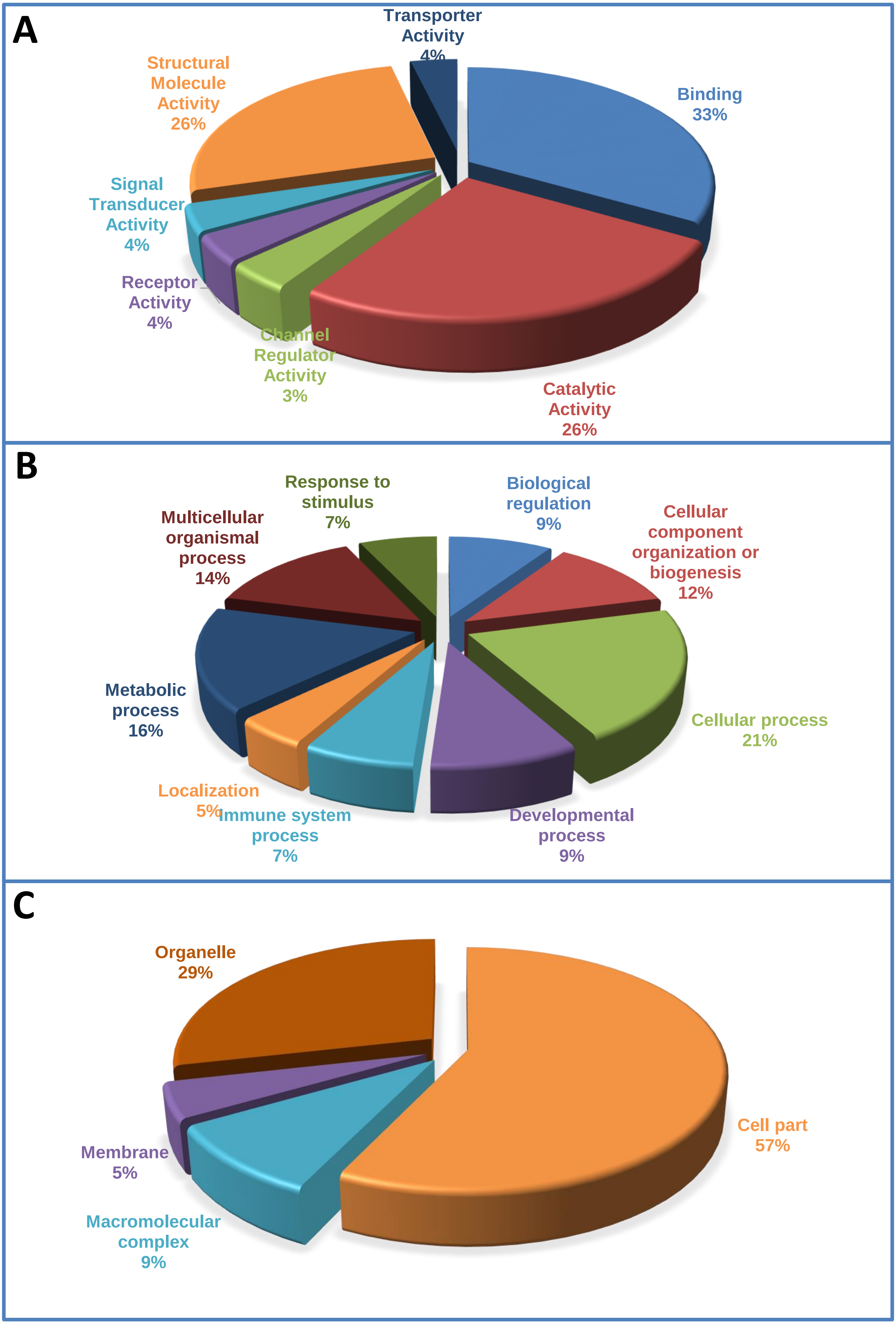
A
Transporter Activity 4%
Binding 33%
Structural Molecule Activity 26%
Signal Transducer Activity 4%
Receptor Activity 4%
Channel Regulator Activity 3%
Catalytic Activity 26%
B
Response to stimulus 7%
Biological regulation 9%
Multicellular organismal process 14%
Cellular component organization or biogenesis 12%
Metabolic process 16%
Cellular process 21%
Localization 5%
Immune system process 7%
Developmental process 9%
C
Organelle 29%
Cell part 57%
Membrane 5%
Macromolecular complex 9%

Figure S5

A

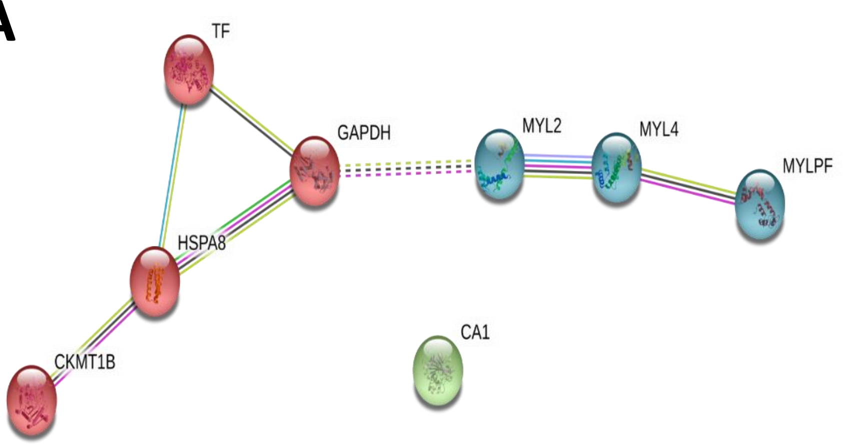
TF
GAPDH
MYL2
MYL4
MYLPF
HSPA8
CA1
CKMT1B

B

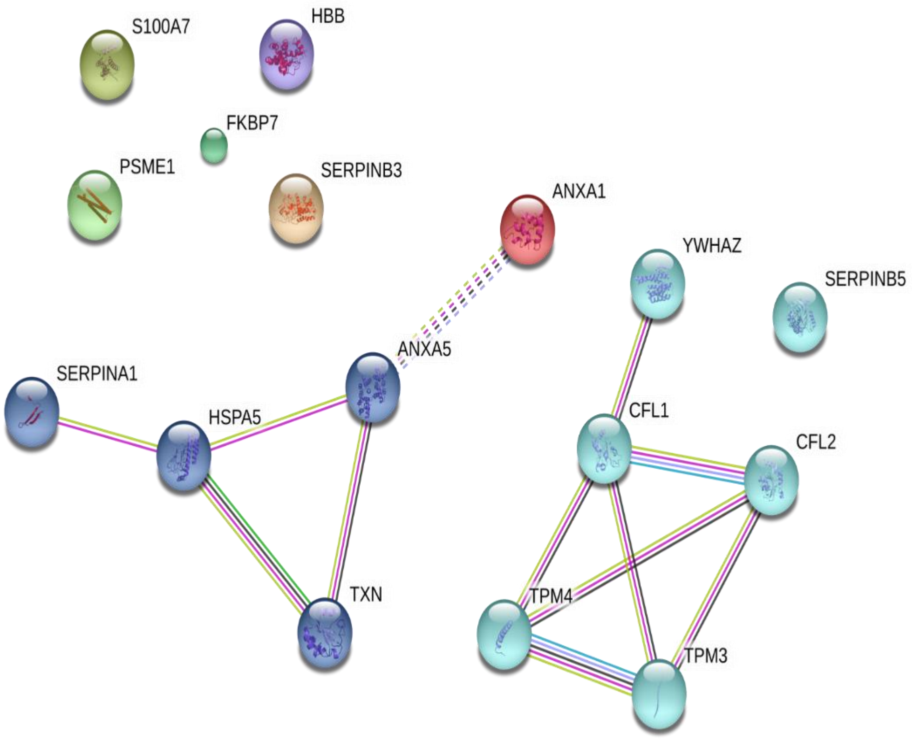
S100A7
HBB
FKBP7
PSME1
SERPINB3
ANXA1
YWHAZ
SERPINB5
ANXA5
SERPINA1
HSPA5
CFL1
CFL2
TXN
TPM4
TPM3

Figure S6

A

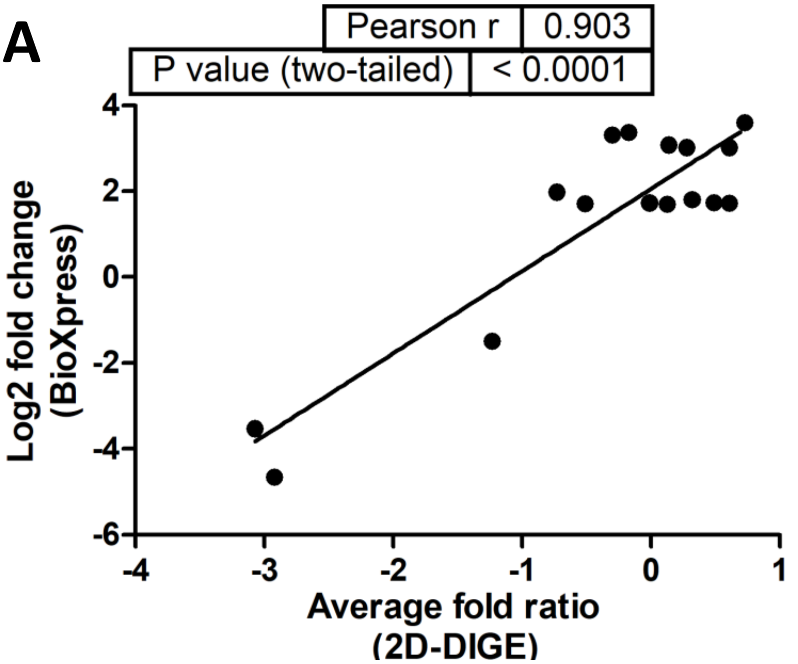

Pearson r | 0.903
P value (two-tailed) | < 0.0001
Log2 fold change (BioXpress)
Average fold ratio (2D-DIGE)
4
2
0
-2
-4
-6
-4
-3
-2
-1
0
1

B

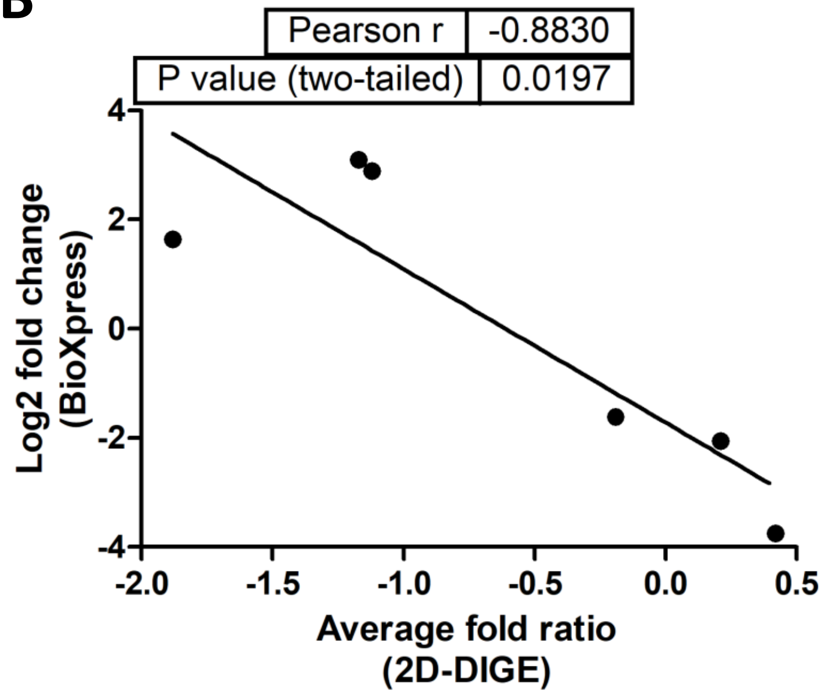

Pearson r | -0.8830
P value (two-tailed) | 0.0197
Log2 fold change (BioXpress)
Average fold ratio (2D-DIGE)
4
2
0
-2
-4
-2.0
-1.5
-1.0
-0.5
0.0
0.5

Supplementary Figure Legends

Figure S1: Representative fluorescent protein profiles of 2D-DIGE containing tongue normal (6 samples) sample labelled with Cy5, tumor sample (6) labelled with Cy3 and pooled internal control labelled with Cy2. Tissue proteins were separated on IPG strip (pH 3–11) in the first dimension followed by 12.5% PAGE in the second-dimension electrophoresis. Images were captured using a Typhoon FLA9500 Variable Mode Imager.

Figure S2: Differential protein expression profile from Tongue tissue samples. Comparison of tongue normal tissue (A) protein profile with tumor tissue (B) samples and identified 24 differentially regulated proteins. Proteins from tissue extracts were separated in 2D-DIGE followed by SDS-12.5% PAGE in the second-dimension. Image was captured using a Typhoon FLA 9500 Imager.

Figure S3: Quantitative Real Time PCR analysis of MMP9 from tongue normal and tumor samples.

Figure S4: Functional classification of identified proteins and biological network analysis. Using PANTHER gene ontology database, 24 differentially regulated proteins were functionally categorized as A- Molecular Function; B- Biological Process; C-Cellular component.

Figure S5: Protein-protein interaction analysis performed using STRING database for eight down regulated proteins (A) and sixteen up regulated proteins (B).

Figure S6: Correlation analysis performed with BioXpress and Average fold-Ratio from 2D-DIGE indicated correlation between the two datasets. **(A)** A positive r-value of Pearson's coefficient indicates a positive correlation observed with 16 genes (YWHAZ, TXN, TPM4, TPM3, SERPINB5, SERPINB3, SERPINA1, S100A7, PSME1, MYLPF, MYL2, HSPA5, FKBP7, CFL1, CA1, ANXA5) whereas **(B)** a negative r-value of Pearson's coefficient indicates a negative correlation observed with 6 genes (MYL4, GAPDH, ANXA1, HBB, CFL2, HSPA8).

Table S1: Patient Demographics used in this study.

.

Description	Discovery Phase (2D DIGE/ nLC MS /MS)	Validation Phase (qRT PCR)	Total
Total patients	30	50	80
Normal/Cancer	30/30	20/30	50/60
Age range (in years)	27-69 yrs	31- 80 yrs	27 – 80 yrs
Mean age (SD) (in years)	40 ±13.1 yrs	48 ±17.6 yrs	43 ±14.2 yrs
No. of males/females	22/8	38/12	60/20

Table S2: The DIGE Experimental Design for Minimal Labelling with CyDyes used in this study.

**Gel No**	**Cy3**	**Cy5**	**Cy2**
1	T14 - 30ug	N14 - 30ug	Pooled Internal standard - 30ug
2	T35 - 30ug	N35 - 30ug	Pooled Internal standard - 30ug
3	N47 - 30ug	T47 - 30ug	Pooled Internal standard - 30ug
4	T68 - 30ug	N68 - 30ug	Pooled Internal standard - 30ug
5	N74 - 30ug	T74 - 30ug	Pooled Internal standard - 30ug
6	N77 - 30ug	T77 - 30ug	Pooled Internal standard - 30ug

T - Tumor; N- Adjacent normal

Table S3: DIA analysis data performed in DeCyder Software for all the six tumor samples. T - Tumor; N- Adjacent normal.

Gel No	Cy3	Cy5	Decreased in tumor	Increased in tumor
1	T14	N14	387	352
2	T35	N35	174	317
3	N47	T47	374	344
5	T68	N68	353	402
4	N74	T74	346	302
6	N77	T77	386	391

Table S4: List of 60 differentially regulated spots which were up regulated in tongue tumor samples describing the protein ID, average fold ratio and statistical significance value.

Protein ID	Av. Ratio	1-ANOVA	Protein ID	Av. Ratio	1-ANOVA	Protein ID	Av. Ratio	1-ANOVA
46	1.6	0.013	20	1.98	0.0045	1	2.47	7.30E-05
122	1.62	0.02	41	1.98	0.012	24	2.51	0.0058
81	1.64	0.046	51	1.98	0.015	112	2.55	0.045
100	1.64	0.036	26	2.02	0.0064	2	2.67	0.00046
61	1.65	0.016	87	2.03	0.029	90	2.73	0.031
37	1.67	0.01	114	2.03	0.046	5	2.8	0.00067
29	1.7	0.0071	64	2.04	0.017	7	2.89	0.00096
60	1.71	0.016	4	2.05	0.00063	97	3.02	0.035
59	1.72	0.016	75	2.07	0.021	40	3.06	0.012
58	1.73	0.016	94	2.12	0.034	82	3.07	0.027
109	1.74	0.043	115	2.12	0.047	39	3.08	0.012
36	1.8	0.01	10	2.13	0.0025	117	3.1	0.048
83	1.84	0.027	86	2.14	0.029	6	3.31	0.0007
42	1.86	0.012	88	2.15	0.03	111	3.55	0.044
14	1.87	0.0035	9	2.17	0.002	25	3.56	0.0062
79	1.9	0.025	121	2.17	0.031	18	3.6	0.0041
45	1.91	0.013	67	2.28	0.018	3	3.75	0.00047
13	1.92	0.0035	19	2.29	0.0043	49	3.9	0.015
27	1.95	0.0068	16	2.3	0.0039	8	4.54	0.0018
68	1.95	0.018	50	2.36	0.015	85	5.28	0.028

Table S5: List of 62 differentially regulated spots which were down regulated in tongue tumor samples describing the protein ID, average fold ratio and statistical significance value.

**Protein ID**	**Av. Ratio**	**1-ANOVA**		**Protein ID**	**Av. Ratio**	**1-ANOVA**		**Protein ID**	**Av. Ratio**	**1-ANOVA**
33	-5.18	0.0082		21	-2.68	0.0046		118	-1.99	0.048
74	-4.74	0.02		96	-2.5	0.034		92	-1.95	0.031
17	-4.68	0.004		73	-2.47	0.02		55	-1.88	0.015
78	-4.65	0.023		56	-2.43	0.015		30	-1.81	0.0073
80	-4.62	0.025		105	-2.41	0.041		98	-1.81	0.035
47	-4.61	0.013		71	-2.4	0.018		113	-1.8	0.045
57	-4.42	0.015		70	-2.35	0.018		23	-1.78	0.0056
44	-4.27	0.012		104	-2.35	0.04		103	-1.77	0.038
34	-3.89	0.0088		72	-2.32	0.02		110	-1.75	0.043
66	-3.77	0.017		101	-2.31	0.036		102	-1.74	0.038
93	-3.75	0.033		106	-2.3	0.042		54	-1.73	0.015
76	-3.64	0.022		12	-2.28	0.0033		65	-1.71	0.017
22	-3.55	0.0048		62	-2.16	0.016		119	-1.66	0.049
120	-3.53	0.05		48	-2.14	0.014		95	-1.64	0.034
63	-3.52	0.016		15	-2.1	0.0035		69	-1.62	0.018
108	-3.48	0.042		32	-2.09	0.0078		31	-1.61	0.0077
99	-3.46	0.035		38	-2.08	0.01		91	-1.6	0.031
107	-3.16	0.042		43	-2.08	0.012		53	-1.53	0.015
84	-3.11	0.027		28	-2.06	0.0068		116	-1.51	0.047
35	-3.08	0.0089		11	-2.04	0.0029		52	-1.49	0.015
89	-2.77	0.03		77	-2.03	0.023				

Table S6: List of primers used for qPCR analysis

Gene	Sequence (5’-3’)
FKBP7(F)	ATGTAGCATCCGGTGGAATC
FKBP7(R)	TGGTGTTGGGCAAGTCATAA
SERPB3(F)	AAGAGCTTGTTGGCGATCTT
SERPB3(R)	AACACCACAGGAAAAGCTGC
SERPB5(F)	AGAGAAGAGGACATTGCCCA
SERPB5 (R)	TTCCTTTTCCACGCATTTTC
TPM3 (F)	CATCTTTTAAGGCCCGGTTT
TPM3 (R)	GCTGAAAGGGACAGAGGATG
TPM4 (F)	TCCAGTGTCTGATGTAAGCCC
TPM4 (R)	ACTGAAAGAGGCTGAGACCC
TXN (F)	TGTTGGCATGCATTTGACTT
TXN (R)	GCCTTGCAAAATGATCAAGC
CKMT1(F)	TCATTCCACATGAACTCCCA
CKMT1 (R)	CCTGATCTGGGTGAATGAGG
MYL2(F)	GTCAATGAAGCCATCCCTGT
MYL2(R)	CTTCCACCATGGCACCTAAG
CA1(F)	CAAACCATCAGCCTTTGAGG
CA1 (R)	CACTGGGGCAGTACAAATGA
YWHAZ(F)	ACAAAAGACGGAAGGTGCTG
YWHAZ (R)	GAAGCATTGGGGATCAAGAA
CFL1(F)	GGTGCTCTTCTGCCTGAGTG
CFL1(R)	TCTTGACAAAGGTGGCGTAG
ANXA5(F)	AAGCCTTTCATAGCCTTCCG
ANXA5(R)	CACAGTCTGGTCCTGCTTCA
TF(F)	CATCCAGTGTCACAGCATCC
TF(R)	CTAAGTGCCAGAGTTTCCGC
CFL2(F)	ATTCTGGGCTCCTGAAAGTG
CFL2(R)	TTCTCTCCAAGTGTCGAACG
PSME1(F)	CGAGCAGGTTCTCTGTCTTG
PSME1(R)	ACTCCACTCCTTGTGCGG
S100A7(F)	TCATCCTTCTACTCGTGACGC
S100A7(R)	CAGGCTTGGCTTGTCAATCT
SERPA1(F)	ACGAGACAGAAGACGGCATT
SERPA1(R)	ATGACTCCTTTCGCAGCCT
HBB(F)	ACCAGCCACCACTTTCTGAT
HBB(R)	AGCTGCACTGTGACAAGCTG
MYLPF(F)	TGAGTCTGGTCGAACATGGA
MYLPF(R)	GGGGACTCCTTGCTTCTTTC
MYL4(F)	CTCGGCATTGGTAGGGTTCT
MYL4(R)	GACTTCACTGCCGACCAGAT
GAPDH(F)	TTAAAAGCAGCCCTGGTGAC
GAPDH(R)	CTCTGCTCCTCCTGTTCGAC
ANXA1(F)	CACCTTTGGATGACTTCACAGT
ANXA1(R)	TCTTTGCAAGAAGGTAGAGATAAAG
HSPA5(F)	TGATTGTCTTTTGTCAGGGG
HSPA5(R)	GGGAGGTGTCATGACCAAAC
HSPA8(F)	CGTGCCATGACAAAGGATAA
HSPA8(R)	TCAACCTCTTCAATGGTGGG
